# Contribution of Keratinocytes in Skin Cancer Initiation and Progression

**DOI:** 10.3390/ijms25168813

**Published:** 2024-08-13

**Authors:** Océane Dainese-Marque, Virginie Garcia, Nathalie Andrieu-Abadie, Joëlle Riond

**Affiliations:** Université de Toulouse, Inserm, CNRS, Université Toulouse III-Paul Sabatier, Centre de Recherches en Cancérologie de Toulouse, 31037 Toulouse, France

**Keywords:** keratinocyte, melanoma, microenvironment, skin carcinoma, tumor invasion

## Abstract

Keratinocytes are major cellular components of the skin and are strongly involved in its homeostasis. Oncogenic events, starting mainly from excessive sun exposure, lead to the dysregulation of their proliferation and differentiation programs and promote the initiation and progression of non-melanoma skin cancers (NMSCs). Primary melanomas, which originate from melanocytes, initiate and develop in close interaction with keratinocytes, whose role in melanoma initiation, progression, and immune escape is currently being explored. Recent studies highlighted, in particular, unexpected modes of communication between melanocytic cells and keratinocytes, which may be of interest as sources of new biomarkers in melanomagenesis or potential therapeutic targets. This review aims at reporting the various contributions of keratinocytes in skin basal cell carcinoma (BCC), cutaneous squamous cell carcinoma (cSCC), and melanoma, with a greater focus on the latter in order to highlight some recent breakthrough findings. The readers are referred to recent reviews when contextual information is needed.

## 1. Introduction

Keratinocytes are the major cellular components of the epidermis, the outer layer of the skin. As the main interface with the external environment, the skin acts as a physical barrier to protect our body from external aggressions, including pathogens, physical agents, chemicals, and ultraviolet (UV) radiation, and prevents dehydration of the underlying tissues. These functions are ensured by a highly organized architecture in which epidermal keratinocytes play a pivotal role. Keratinocytes maintain epidermal homeostasis through reciprocal interactions with neighboring keratinocytes to form an organized and cohesive epidermal structure, with melanocytes to maintain the UV-protective function of the “epidermal unit”, and with immune cells to detect and eradicate pathogen threats. These interactions are finely regulated by cell–cell adhesion, cell-matrix adhesion, and paracrine signaling. Here, we summarize how keratinocytes self-interact and interact with melanocytes within the epidermal microenvironment, and how dysregulations of their cellular functions and interactions contribute to skin carcinomas and melanoma.

## 2. Skin Architecture

**Figure 1:**
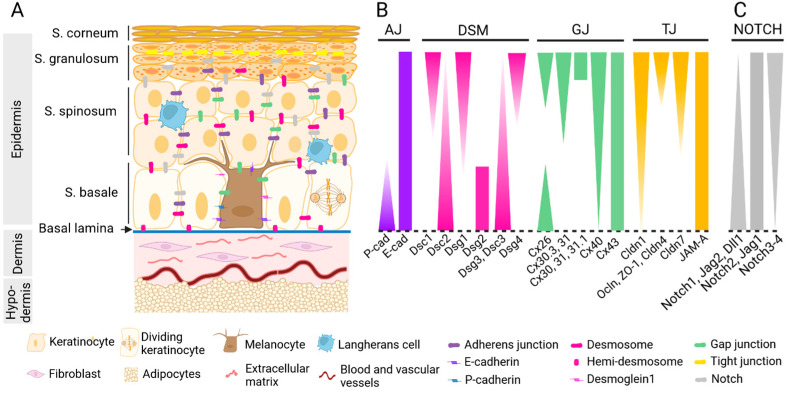
Skin architecture. (**A**) The skin is organized in three superposed layers: the epidermis, the dermis, and the hypo-dermis. (**B**) The epidermis cohesion is ensured by a large repertoire of adhesion molecules variously expressed among the epidermal layers (*Stratum*, S.) and forming different specialized adhesion structures, including adherens junctions (AJs), desmosomes (DSMs), tight junctions (TJs), and gap junctions (GJs). (**C**) Keratinocytes also communicate via Notch signaling.

The skin is organized into three layers: the epidermis and the dermis separated by a semi-permeable basal lamina, and the hypodermis (Figure 1A). The epidermis is a stratified epithelium with adnexal structures (hair follicles, sweat glands, and sebaceous glands) extending to the dermis. Epidermal stem cells continuously self-renew and result in dividing keratinocytes in the basal cell layer (stratum basale). Post-mitotic keratinocytes enter a progressive differentiation program and are pushed upward by newly generated cells, leading to the formation of the squamous cell layer (stratum spinosum), the granular cell layer (stratum granulosum), and the outer cornified or horny cell layer (stratum corneum). The stratum corneum consists of corneocytes and dead keratinized cells embedded in a highly organized lipid matrix, which is an important contributor to the skin-barrier function [1]. Interspersed between dividing keratinocytes in the stratum basale, melanocytes produce and transfer the skin pigment melanin to about 35 surrounding keratinocytes through dendritic extensions, forming the “melanin unit”, which protects the skin from harmful UV radiations [2,3,4]. The epidermis also hosts immune cells, including Langerhans cells and, occasionally, resident T lymphocytes, as well as Merkel cells endowed with mechanoreceptor, neuro-endocrine, and nociceptive functions.

The basal layer of the epidermis is anchored to the basal lamina, which provides mechanical support for epidermal–dermal adhesion and acts as a selective barrier controlling molecular and cellular exchanges with the underlying dermis. The dermis is formed by fibroblasts within a dense collagen-rich extracellular matrix behaving as a structural support and a supply of nutrients for the epidermis. Dermal fibroblasts close to the epidermis secrete Type IV collagen, which is a constituent of the dermal–epidermal junction. Within the dermis, they produce Type I and III collagen and elastin, which provide its mechanical properties and elasticity to the skin. Besides immune cells, mast cells, and macrophages, the dermis encloses blood and lymphatic vascular vessels, sweat and sebaceous glands, hair follicles, nerves, and a variety of sensory nerve receptors. The deepest layer of the skin is the hypodermis, which is predominantly composed of adipocytes, pre-adipocytes, and mesenchymal stem cells, which not only store energy in the form of fat and thermoregulate the body but also protect underlying structures.

## 3. Keratinocyte Interactions with Their Neighboring Keratinocytes and Melanocytes

### 3.1. Interactions via Adhesion

Keratinocytes ensure the epidermal structure and cohesion by expressing a large repertoire of adhesion molecules involved in the formation of various and specialized intercellular junctions, including adherens junctions (AJs), desmosomes (DSMs), gap junctions (GJs), and tight junctions (TJs) [5,6]. This repertoire evolves during the migration of keratinocytes as they progress outwards through the epidermal layers [7] (Figure 1B). Except for E-cadherin, Cx43 and JAM-A are present both in proliferating and differentiated keratinocytes, while adhesion molecules are expressed in a polarized fashion across the multiple epidermal cell layers. This stratification-specific distribution is tied to the keratinocyte-differentiation program [8].

In adherens junctions, adhesion is mediated by the classical members of the calcium-dependent cadherins, E- and P-cadherins. These proteins preferentially bind to the same cadherin proteins on adjacent cells and anchor the actin cytoskeleton to the plasma membrane at sites of cell–cell adhesion via α- and β-catenins. Desmosomes are composed of members of the desmoglein (Dsg) and desmocollin (Dsc) subclasses of desmosomal cadherins. They link the keratin-containing intermediate filaments to the membrane, serve as rigid anchors between adjacent keratinocytes, and tune the cellular and supracellular mechanical properties in the skin [6,9,10]. Whereas Dsg2 expression is restricted to the basal layer, Dsc2, Dsc3, and Dsg3 are expressed in dividing and early differentiating keratinocytes. In contrast, Dsg1 is expressed in keratinocytes only as they transit out of the basal layer and enter differentiation. In addition to their adhesive role, desmosomal cadherins contribute to epidermal homeostasis by coordinating intracellular signaling responses [11]. For instance, Dsg1 facilitates keratinocyte progression through a more differentiated phenotype by dampening EGFR (epidermal growth factor receptor)-Erk1/2 (extracellular signaling regulated by kinase 1/2) signaling [12]. Dsg3 acts as an anti-stress protein by suppressing p53 function responses to stress signals [13].

Gap junctions are specialized channel connections enabling direct intercellular communication between cytoplasms of neighboring cells and are formed by the docking of hemichannels, or connexons, on apposed cell membranes. Connexons are hexamers of transmembrane proteins from the connexin (Cx) family. Gap junctions allow for the exchange of ions, including Ca^2+^, small metabolites, secondary messengers, and short interfering RNAs. In non-junctional areas, hemichannels can mediate the exchange of molecules with the extracellular medium. Up to 10 connexins have been identified in the epidermis, with uneven expression depending on the keratinocyte-differentiation status [14,15]. Keratinocytes from the basal layer express mainly Cx43 and Cx26, while other connexins have a differential expression in stratum spinosum and stratum granulosum. The switch in the gap-junction protein expression in differentiating keratinocytes is accompanied by selective changes in junctional permeability [16] and is associated with the regulation of keratinocyte proliferation and differentiation, in which the Ca^2+^ gradient plays a particular role [14,15].

Tight junctions (TJs) form a strong inside-out barrier in the stratum granulosum 2 layer, which separates the aqueous epidermal environment of the lower layers and the upper physical barrier of the stratum corneum. By sealing the intercellular spaces between adjacent keratinocytes, TJs control the diffusion of water and solutes through the paracellular pathway [17]. Moreover, epidermal TJs regulate the exposure of inside innate immune receptors to their outside ligands, forming an immunological barrier dynamically modulated by the inflammatory milieu [18]. TJs are composed of tetraspan membrane proteins, claudins, and occludin, as well as junctional adhesion molecules such as JAM-A and intracellular TJ plaque proteins including, zonula occludens (ZO), and they are connected with the actin cytoskeleton. In addition to their contribution to barrier formation, some TJ proteins, such as occludin, play a role in cell–cell adhesion and keratinocyte susceptibility to apoptosis induction by UVB or TNF-related apoptosis-inducing ligand (TRAIL) [19].

**Figure 2:**
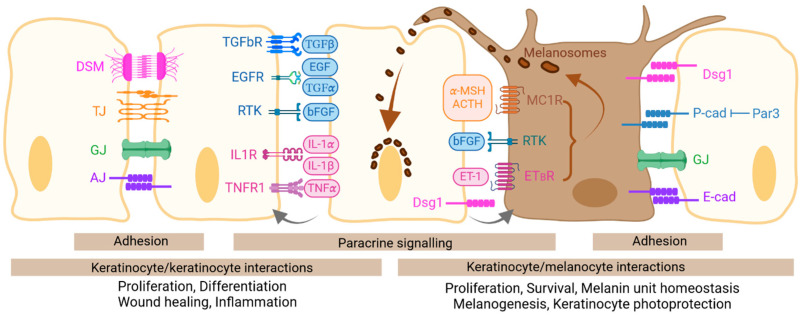
Interactions of keratinocytes with adjacent keratinocytes and melanocytes. Epidermal keratinocytes interact with adjacent keratinocytes (**left**) and melanocytes (**right**) through adhesion-mediated interactions and paracrine signaling. These interactions trigger pathways modulating keratinocyte proliferation, differentiation, wound healing and inflammation, and control melanocyte proliferation and functions.

Basal keratinocytes tightly regulate the growth and behavior of melanocytes through adhesion-mediated interactions [20] (Figure 2). E-cadherin is expressed by both cell types [5] and is the major adhesion molecule controlling keratinocyte–melanocyte interactions [21]. Through this interaction, a ratio of one melanocyte for every 10 keratinocytes is maintained in the epidermal basal layer [22,23]. Moreover, the E-cadherin-mediated interaction controls the phenotype of melanocytes by preventing the expression of melanoma-associated antigens MelCAM and αvβ3 integrin, which are involved in melanoma invasion [23,24]. Melanocyte E-cadherin also mediates UV radiation-induced melanocyte filopodia formation and keratinocyte-contact-dependent melanin transfer [25]. In addition, the heterotypic binding of keratinocyte E-cadherin with αE(CD103)β7 integrin promotes the long-term persistence of αEβ7-expressing immune cells in the skin [26]. Basal keratinocytes and melanocytes also express P-cadherin. Its expression on keratinocyte surfaces is controlled by the epidermal polarity protein Par3, which limits surface P-cadherin by regulating its turnover [27]. Homotypic transcellular interaction stabilizes P-cadherin in melanocytes and controls their growth, differentiation, and motility. Par3 loss in keratinocytes increases P-cadherin-based adhesion with melanocytes, eliciting melanocyte proliferation and a phenotypic switch toward dedifferentiation [27].

Although there are no desmosomes present in melanocytes, Dsg1 is expressed in melanocytes and is downregulated during melanomagenesis [28]. Dsg1 has a similar expression pattern to E-cadherin and is a co-receptor for E-cadherin [28]. Moreover, Dsg1-expressing keratinocytes control the proper localization of melanocytes in the basal layer, and their pigmentation and dendrite morphology by a Dsg1-dependent paracrine signaling [29]. 

Melanocytes express the connexins Cx26 and Cx43, which enable heterocellular gap-junctional intercellular communication (GJIC) with keratinocytes [30,31], possibly regulating melanocyte growth through GJIC [22].

The loss of E-cadherin and Dsg1 during melanomagenesis disrupts the keratinocyte control over melanocytic cells and is associated with GJ incompatibility [30]. The restoration of E-cadherin [23], Dsg1 [28], or Cx43 [32] expression was shown to reduce melanoma growth.

### 3.2. Interactions via Notch Signaling

Notch signaling constitutes an essential form of intercellular communication between cells and is decisive in the development and homeostasis of multiple tissues [33]. The canonical Notch signaling involves the interaction between Notch ligands, including delta-like ligand 1 (DLL1), delta-like ligand 3 (DLL3), delta-like ligand 4 (DLL4), Jagged-1 (JAG1), and Jagged-2 (JAG2) on signal-sending cells and Notch receptors Notch1–4 on signal-receiving cells. The ligand-receptor interaction leads to the cleavage of the Notch intracellular domain (NICD), its translocation into the nucleus, and its interaction with the DNA-binding protein RBP-Jκ (CSL). The formed complex recruits the co-activator Mastermind (Mastermind-like (MAML) 1–3 in vertebrates), which activates the transcription of target genes, including the Notch target-gene families Hairy/Enhancer of Split (HES) and Hairy/Enhancer of Split related to YRPW motif (HEY).

Notch signaling is involved in epidermis differentiation [34]. Keratinocytes in the basal layer express DLL1, JAG2 ligands, and the Notch1 receptor, whereas keratinocytes, which initiate and undergo terminal differentiation in suprabasal layers, express JAG1 and Notches1–4 (Figure 1C). Activated Notch1 causes keratinocyte growth arrest by inducing p21WAF/Cip1 expression through RBP-Jκ-dependent transcription, whereas activated Notch1 and Notch2 promote the expression of differentiation markers, such as keratin 1 and involucrin, in an RBP-Jκ-independent pathway [35]. Activated Notch1 expression also downregulates α3/β1 and α6/β4 integrin expression, reducing keratinocyte adhesion to the substratum [35]. Moreover, it negatively regulates Wnt signaling through downmodulation of the Wnt gene expression, restricting keratinocyte proliferation [36].

### 3.3. Interactions via Paracrine Signaling

Keratinocytes secrete several classes of molecules, including cytokines, growth factors, antimicrobial peptides, enzymes, neurothrophic factors, and non-protein molecules/products involved in skin homeostasis, regeneration, defense, and inflammation [37,38]. Transforming growth factor (TGF) family members are constitutively expressed in the epidermal basal layer and are involved in wound repair by controlling keratinocyte proliferation and differentiation [39]. Other factors of the epidermal growth factor (EGF) and fibroblast growth factor (FGF) families, such as EGF, TGFα, bFGF, and the neurotrophic peptide NGF, promote keratinocyte proliferation and migration [40,41,42]. Keratinocytes also produce pro-inflammatory cytokines, such as IL-1α, IL-1β, IL-6, and TNFα, which increase their proliferation and migration [42,43].

Factors secreted by keratinocytes also control melanocyte proliferation, adhesion to the basal lamina, survival upon stress exposure, genomic stability, and the ability to synthesise and transfer melanin [44,45,46] (Figure 2). Keratinocytes produce bFGF [47], endothelin-1 (ET-1) [48], and bioactive products of proopiomelanocortin (POMC), α-MSH, adrenocorticotropic hormone (ACTH), and β-endorphin, which exert mitogenic effects on melanocytes through binding to their respective receptors. The exposure of keratinocytes to UV upregulates the synthesis of bFGF, as well as IL-1α, which in turn upregulates αMSH and ET-1 synthesis by keratinocytes. Keratinocyte-secreted ET-1 can downregulate E-cadherin on melanocytes [49], facilitating their decoupling from keratinocytes before division. Moreover, ET-1 upregulates MelCAM in melanocytes [50]. In addition to their mitogenic effects on melanocytes, α-MSH and ACTH are key inducers of melanogenesis and of the dendrite formation for melanin delivery to the connected keratinocytes [51,52,53]. Through their binding to MC1R, they stimulate eumelanin synthesis by increasing the microphthalmia-associated transcription factor (MITF), tyrosinase, and tyrosinase-related protein (TRP) 1 levels, as well as the activity of tyrosinase [51,54,55]. Melanin is then transferred to keratinocytes in which it accumulates in the supra-nuclear region, protecting the nuclear genetic material from UVR-induced damage [56].

## 4. Role of Keratinocytes in Non-Melanoma Skin Cancers

The oncogenic transformation of keratinocytes initiates the development of skin carcinomas, which are the most common cancers in Caucasian populations [57]. Also called non-melanoma skin cancers (NMSC), they include basal cell (BCC) and cutaneous squamous cell carcinomas (cSCC), which account for about 80% and 20% of skin carcinomas, respectively. These cancers are mostly caused by UV radiation but are distinct tumor entities. BCCs grow locally in the epidermis, sometimes aggressively with extensive tissue destruction. Superficial and nodular BCCs are considered low-risk tumors, whereas infiltrative BCCs have a higher risk of progression, including metastasis in less than 0.04% of BCC cases [58]. cSCCs are usually associated with advanced age and an increasing risk with older age, and they can metastasize primarily to the lymph nodes in only 5% of patients with advanced skin lesions, with a 5-year survival rate of 25–50% in this case [59].

### 4.1. Basal Cell Carcinoma

BCCs develop from stem cells of the epidermis, hair follicles, and eccrine sweat in permissive skin areas [60] and are mostly driven by aberrant activation of the Hedgehog (Hh) pathway [61]. Hh signaling is involved in embryonic skin and hair follicle development, as well as in epidermal stem cell maintenance [62]. In the absence of Hh family ligands, the Patched-1 (PTCH1) receptor inhibits the downstream protein Smoothened (SMO). Upon ligand binding or mutation inactivating PTCH1, SMO inhibition is released, leading to a dissociation of the repressor Sufu from Gli transcription factors. The translocation of Gli proteins into the nucleus induces transcription of Hh target genes and promotes proliferation and tumor growth [63]. Whereas low-risk or superficial BCCs are mostly managed by surgery, locally advanced or rare metastatic BCCs (mBCCs) are treated with the specific SMO inhibitors vismodegib and sonidegib [64].

**Figure 3:**
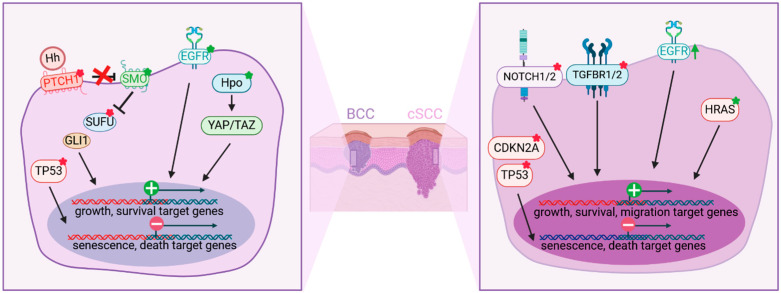
Main genetic deregulations in basal cell carcinoma and cutaneous squamous cell carcinoma. (**Left**) BCC is mostly driven by mutations leading to aberrant activation of the Hedgehog (Hh) pathway. Additional activating mutations in the EGFR and Hippo (Hpo) pathways contribute to the expression of growth and survival target genes. Inactivating TP53 mutation promotes resistance to senescence and cell death. (**Right**) cSCC presents mainly inactivating mutations in TP53 and CDKN2A promoting resistance to senescence and cell-death signals. Inactivating mutations in Notch1 and TGFBR1/2, activating mutations in HRAS, and increased expression of EGFR promote growth, survival, and migration. Inactivating or activating mutations are indicated with a red or green star, respectively. Increased expression is indicated with a green arrow.

Early studies identified germline mutations in PTCH1 that are associated with Gorlin syndrome, an inherited disease predisposing to early onset of BCCs [65], and they found PTCH1 mutations in sporadic BCCs [66]. A wide description of the genetic basis of BCCs by whole exome studies from large BCC cohorts confirmed the predominance of inactivating PTCH1 mutations in sporadic BCCs (in 73% of cases) and identified activating mutations in SMO (20%) (Figure 3). A small fraction of tumors (8%) harbor loss-of-function mutations in the negative regulator of the Hh pathway SUFU [67,68]. BCCs (61%) also present somatic inactivating mutations in TP53 gene that are known to be involved in keratinocyte senescence [69]. Additional events, including driver mutations in other cancer-related genes such as members of the Hippo-YAP, MYCN/FBXW7, EGFR, phosphatidylinositol 3-kinase (PI3K)/protein kinase B (AKT) pathways, and members of the protein kinase C (PKC) family, were also found in BCCs and contribute to BCC tumorigenesis and phenotypic diversity [61,67].

Several studies reported the differential expression of regulatory non-coding RNAs (ncRNAs), including microRNAs (miRNAs) and long non-coding RNAs (lncRNAs), in BCCs compared to normal skin and among BCC subtypes [70,71,72]. For instance, the expression of miRNA-203, which acts as a tumor suppressor in BCC, is decreased upon the activation of the Hh pathway. This contributes to the oncogenic transformation of BCC via the derepression of multiple stemness- and proliferation-related genes [73]. MiRNA-183, which inhibits cell migration and invasive behavior in vitro in various cancer cell lines, has a reduced expression in infiltrative BCCs compared to nodular BCCs [74]. In contrast, various miRNAs are upregulated and can act as oncomiRs [70,71,72]. The potential of selected miRNAs as therapeutic targets or biomarkers is currently being explored [75,76].

Whole-genome RNA sequencing (RNA-Seq) analyses confirmed the transcriptional upregulation of Hh downstream targets and genes of the Wnt-signaling pathway [77] and identified additional pathways implicated in BCC tumorigenesis, progression [67,78,79], and resistance to treatments [67,78,80,81]. Noticeably, the Hippo-YAP pathway and N-Myc target genes are significantly upregulated in BCC [67], as well as IL17 pathway targets, including cytokines such as CXCL9 and matrix metalloproteinases involved in tumoral inflammation, such as MMP1 and MMP10 [78]. Other significant modulations were found in pathways as diverse as TLR, Akt/PI3K, cadherins, integrins, and PDGF [78].

### 4.2. Squamous Cell Carcinoma

cSCCs are frequently found in UV-exposed regions such as the head and neck (HNSCCs). Besides UV radiation, which is the major cause for the high mutational burden in cSCCs, risk factors also include immunosuppression, infections, specific drugs, and exogenous chemical mutagens, as well as chronic conditions such as cutaneous inflammation and genetic predispositions such as repair-deficient genetic backgrounds [82,83].

cSCCs arise from progenitor cells within both the hair follicle and the interfollicular epidermis. They can progress from different types of premalignant lesions, including actinic keratosis (AK) in 60% of cases [84,85], even though high-risk tumors are often not derived from precursor lesions [86]. AK lesions are characterized by perturbed epidermal differentiation with cellular atypia. They either spontaneously regress, persist, or progress to cSCC in situ (cSCCis) or invasive cSCC, the latter being associated with a significant risk of high recurrence, peri-neural invasion, and loco-regional metastasis [87].

AK and the progression to cSCC involve cumulative mutations that lead to the disruption of the cell cycle and differentiation-program control, as well as aberrant interactions with the tumor microenvironment [83]. Among the high background of UV-induced genetic lesions, mutations in TP53, NOTCH1/2, CDKN2A, and HRAS [88] are found in precancerous cells [89] and persist through clonal expansion in established tumors, suggesting their driver role in carcinogenesis (Figure 3). The loss of function of the tumor-suppressor gene TP53 is detected in 54–95% of SCC cases [90] and is associated with UV signature mutations [91,92]. Clones with heterozygous TP53 mutations are present in keratinocytes of sun-exposed normal skin and can expand upon ongoing UVB exposure, allowing for the accumulation of further genetic damage and uncontrolled proliferation of keratinocytes [93,94]. The complete inactivation of the p53 function, which precedes the expansion of mutations and the development of chromosomal aberrations, is more frequent in invasive diseases [93]. Subsequent genetic alterations leading to unrestrained cell cycling and uncontrolled cell growth are found in the tumor-suppressor locus CDKN2A (76% of the cases) [95]. NOTCH1/2 gene mutations, which are found in 75% of cases [96,97], disrupt the balance between keratinocyte growth and differentiation [98] and contribute to establish a pro-tumoral microenvironment [99]. The oncogene HRAS, rather than NRAS and KRAS, is mutated in cSCCs [100], particularly in cSCCs occurring in melanoma patients treated with BRAF inhibitors (3–20%) [101,102], and it promotes the upregulation of downstream MAPK and PI3K/AKT/mTOR signaling. TGFβ-signaling inactivation by mutations in both the TGFβ Type 1 receptor (TGFBR1) and TGFβ Type 2 receptor (TGFBR2) genes, found in 43% of cSCCs, might be a cooperating event in cSCC development [103]. Additional gain- or loss-of-function mutations and epigenetic dysregulations [71,83] contribute to intra-tumor heterogeneity and discriminate moderately and poorly differentiated cSCCs, which have a worse prognosis, from well-differentiated tumors [104]. The EGF receptor (EGFR), frequently overexpressed in high-risk (~35%) or metastatic cSCC (~80%), is associated with a poor prognosis [105]. EGFR signaling activates RAS/RAF/MEK/MAPK, PI3K/AKT/mTOR, phospholipase C, STAT, and NF-κB pathways [106] and promotes proliferation, migration, survival, resistance to senescence, and altered differentiation [107]. Ineligible candidates for PD-1 inhibitors (cemiplimab and pembrolizumab) with unresectable, locally advanced, or metastatic cSCCs are treated with EGF receptor inhibitors (cetuximab) combined with chemotherapy or radiation therapy [82,108].

The differential regulation of ncRNA was reported in cSCC [70,71,72,109]. As an example, the long intergenic non-coding RNA (lincRNA) PICSAR, which is the most expressed lncRNA in cSCC compared with normal keratinocytes, promotes the growth of cSSC cells by activating the ERK1/2 pathway [110] and migration by downregulating α2β1 and α5β1 integrin expression [111]. The PRECSIT lncRNA expression, which is physiologically repressed by p53, is elevated in cSCC tumors, as compared with cSCCis and AK. Its overexpression promotes the progression of cSCC via STAT3 signaling [112]. The interest of ncRNAs as diagnosis and prognosis biomarkers is currently being investigated [113,114].

### 4.3. Epidermal Microenvironment of BCC and cSCC

Skin cells harbor numerous cancer-related mutations with no change in their physiological functions. The triggering of oncogenic progression is often associated with the loss of homeostasis of the epidermis microenvironment under various conditions [115].

Early histologic and immunohistochemical studies of BCCs and SCCs indicated several epidermis abnormalities in the proximity of carcinomas. Markers of the terminal keratinocyte differentiation normally expressed in the upper layers of the epidermis are greatly decreased in BCCs and cSCCs. The expression of these antigens in adjacent keratinocytes was found to gradually decrease with increasing proximity to the BCCs [116]. The expression of involucrin, which is produced in the outer squamous cell layers of healthy skin and contributes to the formation of the stratum corneum, is also modified in non-neoplasic epidermis adjacent to skin carcinomas. The epidermis layers overlying BCCs show an abnormal pattern of expression, extending to the site of attachment between BCC and the overlying epidermis. In SCCs, the adjacent epidermis displays an increased staining of involucrin extending to the basal layer [117].

Some alterations were found in the expression of adhesion molecules. BCCs and SCCs display heterogeneous staining of the gap junction connexins Cx26 and Cx30, which are usually not detectable in normal epidermis [118]. In particular, SCCs show Cx26 and Cx30 labeling in differentiated areas but weak reactivity in dedifferentiated, i.e., more invasive areas. Strong labeling for Cx26 and Cx30 was also found in keratinocytes adjacent to BCC compared to the distal epidermis. In SCC, where a clear demarcation of tumor and the adjacent epidermis is lacking, the highly differentiated regions at the margin of SCC, which resemble normal epidermis, were usually positive for Cx26 and Cx30.

Nodular and superficial BCCs display a complex pattern of E-cadherin and P-cadherin expression. Most infiltrative BCCs have a reduced E-cadherin expression with no P-cadherin change. Interestingly, whereas normal epidermis expresses P-cadherin in the basal layer and E-cadherin in every epidermal layer with a stronger expression in the spinous layer, the anomalous P-cadherin expression and reduced E-cadherin expression in the spinous layer of skin overlying infiltrative BCC were reported [119]. Moreover, an immunohistochemical analysis of the normal-appearing epidermis overlying basal cell carcinomas revealed an increased proliferative index compared with the epidermis overlying or adjacent to SCC or normal skin. In addition, EGFR, normally restricted to basal and suprabasal layers, was detected in all layers, basal cell keratins were detected in the uppermost layers and basement-related antigens were decreased [120].

These modifications, some of them being also encountered in various benign epidermal hyperplasia [117,118], indicate important phenotypic changes in keratinocytes surrounding carcinoma lesions. It was suggested that they might be either features of a preneoplastic status or reactions to the tumors through diffusible factors produced by malignant cells and/or activated stromal cells. Interestingly, cancer-associated fibroblasts (CAFs) isolated from the TME of BCC influence the in vitro growth and differentiation pattern of normal keratinocytes in co-culture, in a paracrine manner [121]. The secretome of squamous carcinoma cells A431 was shown to induce significant changes in the HaCaT keratinocyte transcriptome and promote their proliferation and migration, in part by the activation of mTOR signaling in keratinocytes [122].

Recent studies using single-cell multi-omic technologies coupled with multiplexed imaging tools refined the NMSC trajectories by integrating the identification of the cellular constituents combined with their transcriptional reprogramming and potential crosstalks within their spatially organized landscape. They revealed the Activin A-mediated dialogue between BCC and extracellular matrix (ECM)-remodeling fibroblasts in the invasive niche [123], the role of TREM2+ skin cancer-associated macrophages (SCAMs) in supporting BCC proliferation [124], and the developmental signature of fibroblasts and pericytes associated with BCCs [125]. In cSCC, a tumor-specific keratinocyte (TSK) population with invasive and immunosuppressive features was identified at the leading edges [126]. Nevertheless, whereas the discrimination of transformed versus non-tumoral keratinocytes is technically achievable [123,125,127], the profiling of carcinoma-adjacent epidermis in comparison with distal healthy epidermis was not yet reported. This may bring additional clues to the comprehension of early skin carcinoma progression.

## 5. Role of Keratinocytes in Primary Cutaneous Melanoma

### 5.1. Primary Melanoma

Primary cutaneous melanomas originate from melanocytes and evolve through distinct trajectories, depending on their anatomical site of origin, degree of cumulative exposure to UV radiation, host age, mutation burden, and types of oncogenic alteration [4]. Oncogenic transformation is associated with recurrent and sequential mutations in key signaling pathways. Melanocytic proliferation initiates with mutations in BRAF, NRAS and NF1 genes, which activate the MAPK signaling pathway. In normal nevi, melanocytic BRAF^V600E^-induced proliferation is controlled by oncogene-induced senescence (OIS) [128], but approximately 30% of melanomas progress from pre-existing/adjacent BRAF^V600E^ nevi acquiring secondary mutations [129]. Most melanomas arise de novo from fields of melanocytic atypia, particularly in sun-damaged skin, and are associated with BRAF^nonV600E^ (such as BRAF^V600K, K601E, G469A^), NRAS (NRAS^Q61R, Q61K^), and NF1 mutations. Invasive primary melanomas and advanced stages display additional alterations in pathways involved in the replicative lifespan (TERT promoter), cell cycle control (CDKN2A), cell identity (ARID2), growth and metabolism (PTEN), and resistance to apoptosis (TP53) [4].

The earliest signs of oncogenic transformation include increased melanocyte density, changes in cellular features, and movement away from the dermal–epidermal junction [130,131]. These precursor lesions can develop into melanoma in situ (Mis) through a pagetoid growth with superficial spreading for BRAF^V600E^ cells or through a lentiginous growth with confluent individual melanocytes along the dermo–epidermal junction for BRAF^nonV600E^ cells, leading to radial growth phase (RGP). The RGP phase can display some features of invasion in the papillary dermis with no competency to form metastasis. In contrast, invasive and highly mitotic growth into the lead to vertical growth-phase (VGP) melanoma with a high potential for metastasis [132,133]. This phase is associated with an epithelial-to-mesenchymal-like transition or phenotypic switching of melanoma cells triggered by genetic events and microenvironmental contexts, ultimately promoting tumor heterogeneity [134,135,136].

### 5.2. Melanoma Induces Deep Changes in the Architectural Features of Epidermis

Contrasted features of epidermis changes in the vicinity of melanoma were reported. Pseudoepitheliomatous hyperplasia (PEH), a reactive epithelial proliferation associated with infectious, inflammatory, and neoplastic conditions, was sporadically described in melanoma [137,138,139]. Drunkenmolle et al. depicted paratumoral epidermal hyperplasia (PTEH) along the lateral borders of the melanoma [140]. This feature was found with a high incidence in thick melanomas of patients who developed no further metastases, suggesting a protective effect of deep-penetrating PTEH. In the case of nodular melanoma, an increased thickness of the epidermis was reported in 90% of biopsies and was associated with aberrant suprabasal expression of keratin 14 (KRT14) in the proximal epidermis [141].

Epidermal hyperplasia was reproduced in nude mice by injection of conditioned media from human melanoma cell lines into the dermis, suggesting the involvement of soluble mediators produced by melanoma cells [142]. In this study, the authors reported the correlation between the hyperplasia extent and the melanoma production of TGFα, known to stimulate the proliferation of keratinocytes. In addition, hyperplasia of the epidermis immediately overlying the tumors (at least 1.0 mm in depth) was associated with an increase in microvessel density (MVD), i.e., increased angiogenesis and a decreased expression of IFN-β, an endogenous anti-angiogenic molecule produced by differentiated keratinocytes [142].

In vitro co-culture experiments of human primary keratinocytes (HPK) and melanoma cells in separated chambers reproduced the increased expression of KRT14 in HPK, demonstrating the involvement of paracrine signaling in HPK-differentiation control. FGF-2, CXCL-1, IL-8, and VEGF-A were identified as mediators of the activity of melanoma cells on keratinocyte phenotype [141].

In contrast, the thinning of the basal and suprabasal epidermal layers and loss of rete ridges in areas of direct contact with neoplastic melanocytes, a phenomenon also known as consumption of the epidermis (COE) or epidermal effacement, was reported in melanomas with aggressive histopathologic features. COE was associated with highly proliferating tumors and was proposed as the first step of progression to ulceration [143]. COE was frequently detected in invasive melanomas and was correlated to increased Breslow depth. The absence of lymphocytes and necrotic keratinocytes in areas of consumption suggests that COE results from mechanical displacement of keratinocytes during melanoma growth rather than inflammatory destruction of keratinocytes [144]. However, epidermal effacement is found in some melanomas with a low Breslow depth, suggesting additional mechanisms such as the action of metalloproteases or alteration of adhesion [145].

Moreover, an analysis of melanocytic lesions using in vivo confocal laser scanning microscopy (CLSM) revealed a loss of the characteristic honeycomb pattern associated with a healthy epidermis. Pagetoid infiltration of melanocytic cells was associated with a disarranged pattern in superficial layers of the epidermis and poorly defined or absent keratinocyte cell borders [146,147,148], suggesting a deregulation of keratinocyte adhesion. Of note, epidermal areas surrounding invasive primary melanoma have a reduced expression of Par3 associated with an increased expression of P-cadherin at contact sites between keratinocytes and melanocytes. In particular, P-cadherin is detected along melanocyte dendrites, protruding towards upper keratinocytes in Mis [27].

Interestingly, the search for biomarkers refining American Joint Committee on Cancer (AJCC) low-stage melanoma classification among autophagy-related proteins pointed out the variable expression of activating molecules in Beclin 1-regulated autophagy protein 1 (AMBRA1) in the epidermis overlying primary melanomas [149]. The loss of peritumoral AMBRA1 identifies AJCC Stage I melanomas with high metastatic risk. Mechanistically, tumor paracrine TGFβ2 signaling decreases the expression of AMBRA1 in keratinocytes and disrupts epidermal integrity, facilitating tumor ulceration and/or metastasis [150]. The combination of AMBRA1 and loricrin expression represents an independent prognostic biomarker in non-ulcerated AJCC Stage I and II melanoma, which can stratify patients at a low risk of disease recurrence [151].

In 10–35% of cases, cutaneous primary melanomas regress spontaneously with partial or complete disappearance of the tumor upon immune intervention [152]. Histology reveals complete or partial replacement of the tumor by vascular fibrous tissue, inflammatory infiltrate, pigment-laden macrophages, blood vessels, epidermal attenuation, and/or apoptosis of keratinocytes or melanocytes. The prognostic significance of melanoma regression remains controversial [153]. Whereas regression in thin primary cutaneous melanoma is associated with a good survival outcome, the presence of tumor regression predicts the lowest overall and relapse-free survivals among patients whose tumors had already spread to sentinel lymph nodes [154].

**Figure 4:**
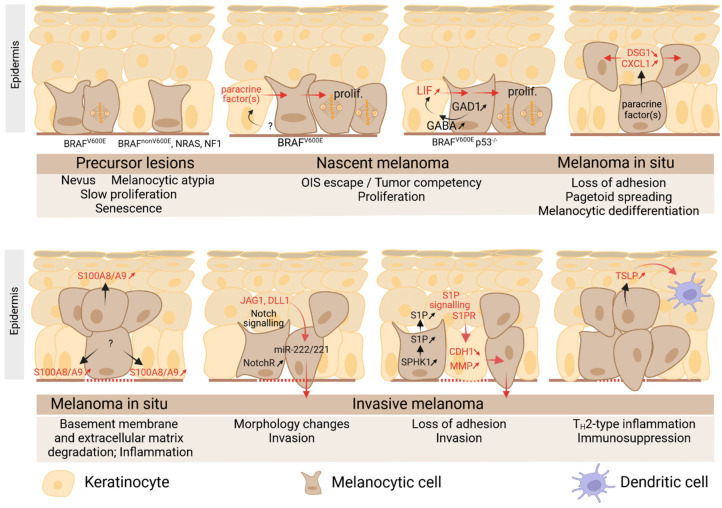
Recent advances in the comprehension of keratinocyte contributions to melanoma initiation and progression. Molecular events attributed to keratinocytes and functional consequences are reported in red.

Taken together, these studies indicate that melanomas induce profound stresses in the surrounding epidermis. As major components of the skin barrier against different external aggressions, keratinocytes display various skills as stress sensors and responders. How such skills are engaged in the case of melanoma aggression, and how this impacts melanoma progression, was recently reviewed [155] and will be discussed in the following sections with a focus on recent discoveries (Figure 4).

### 5.3. Role of Keratinocytes in Melanoma Initiation

Employing microfluidics to co-culture multiple skin cell types in a desired pattern, Sadangi et al. [156] revealed that keratinocytes suppress oncogene-induced senescence (OIS) in BRAF^V600E^ melanocytes in a paracrine signaling-dependent manner. In the presence of keratinocytes, BRAF^V600E^ melanocytes downregulate the expression of cell cycle regulatory genes known as tumor suppressors (CDKN1B, CDKN1C, CDKN2A, and CDKN2C, which code for p27, p57, p16, and p18, respectively), and of genes associated with senescence (CALR1, ETS1, and EGR1). In contrast, it upregulates MAPK14, a component of the MAP kinase pathway associated with an increased cell proliferation and THSB1 coding for thrombospondin, which has been shown to regulate OIS escape and also contribute to a more invasive phenotype of melanoma.

More recently, keratinocytes were also involved in melanoma initiation by BRAF^V600E^. Using an elegant genetic reporter system in zebrafish and in vitro co-cultures, Tagore et al. [157] revealed a GABA-mediated communication from nascent melanoma cells (melanocytes with BRAF^V600E^ and p53^−/−^ alterations) to adjacent keratinocytes, through specialized inhibitory electrochemical cell–cell junctions. Importantly, some of the “switched” keratinocytes, i.e., those in direct physical contact with melanoma, are endowed with protumorigenic properties. Mechanistically, BRAF^V600E^ melanoma cells were found to overexpress the GABA-synthesizing enzyme GAD1, as compared to melanocytes and to produce GABA. By acting on GABA-A receptors in keratinocytes, GABA activates GABAergic signaling, triggers the keratinocyte chloride efflux, and inhibits electrical signaling between melanoma cells and keratinocytes. Importantly, the genetic or pharmacological disruption of GABAergic signaling either in melanoma cells or keratinocytes abrogates this communication. The major role of the GABAergic pathway in the development of nascent melanoma cells was shown in zebrafish and mice, as well as in a human reconstructed skin model. Furthermore, the activation of GABAergic signaling was detected in keratinocytes proximal to tumor cells in Mis. GABA-A receptor–positive keratinocytes were found to mediate their protumorigenic effect by upregulating MYCN, a known upstream activator of LIF expression, and by secreting LIF, which in turn increases melanoma cell proliferation. Whether this cascade depends on the inhibition of electrical activity per se remains to be understood. Nevertheless, the involvement of MYCN, which is associated with early skin development in mammals and which is expressed in keratinocytes [158,159], suggests that GABAergic signaling in melanoma cells may activate an early developmental program in keratinocytes that supports melanoma growth.

Since the upregulation of GAD1 in human melanoma biopsies correlates with primary tumor-forming ability, GAD1 expression might be a useful prognostic marker to predict early tumor initiation from pre-cancerous moles. Furthermore, modulating this communication could open new pharmacological strategies.

### 5.4. Role of Keratinocytes in Melanoma Early Pagetoid Movement

Burks et al. recently revealed a pivotal role for keratinocyte Dsg1 loss in the initiation of melanoma cell movement within their primary epidermal niche [160]. Starting from previous observations showing that the long-term loss of keratinocyte desmoglein 1 (Dsg1) promotes pagetoid movement of melanocytes, an upward intraepidermal spread that mimics a malignant phenotype in 3D organotypic cultures [29], the authors [160] examined Dsg1 expression in the epidermal niche of melanoma cells. Interestingly, Dsg1 is decreased in keratinocytes adjacent to malignant compared to benign melanocytic lesions in clinical samples, and in 3D-organotypic co-cultures mimicking melanocytic foci in a stratified epidermis. Furthermore, in vitro treatment of differentiated keratinocytes with melanoma, but not melanocyte-derived conditioned media, reduced Dsg1 mRNA and protein levels in keratinocytes, indicating that melanoma downregulates keratinocyte Dsg1 through paracrine signaling. Decreased Dsg1 expression is associated with a negative enrichment of gene sets characterizing the most differentiated keratinocyte states. In vitro experiments showed that melanoma-conditioned media induce upregulation of the transcriptional repressor Slug in keratinocytes and consequently decreased the expression of the transcription factor Grainyhead like 1 (Grhl1), a transcriptional activator of Dsg1. Importantly, media from Dsg1-deficient keratinocytes increased melanoma cell migration in trans-well migration assays and melanoma cell migration exhibited a significant negative correlation with Dsg1 expression levels in keratinocytes. In addition, genes enriched in the melanoma cells treated with shDsg1 keratinocyte-conditioned media include genes related to extracellular matrix (ECM), adhesion, and migration. The involvement of keratinocyte Slug/Grhl1/Dsg1 pathway activation in melanoma cell movement is supported by patients’ samples analysis where keratinocyte Dsg1 and Slug were found negatively and positively correlated, respectively, to the extent of intraepidermal melanoma spread.

Whereas the melanoma factors causing this signaling cascade in keratinocytes are not yet identified, Burks et al. showed that keratinocytes treated with melanoma-conditioned media secrete CXCL1, an inflammatory chemokine whose secretion relies on the activation of the ERK1/2 pathway, and which promotes melanoma cell migration via the CXCR2 receptor in melanoma cells.

Taken together, these results highlight the role of keratinocytes in the early steps of intra-epidermal melanoma migration, independently of any detectable decrease in E-cadherin expression, showing that melanoma epidermal spread is not an indirect result of loss of this classical cadherin. In addition to its potential role as an adhesion molecule, Dsg1 plays a pivotal role in this phenomenon as a regulator of paracrine signaling.

Interestingly, melanoma cells treated with media from Dsg1-deficient keratinocytes display an enrichment in a neural crest-like signature and a negative enrichment of the melanocytic signature, suggesting a phenotypic switch toward a dedifferentiated status in agreement with the pro-inflammatory effect of Dsg1 loss [29,161,162].

### 5.5. Role of Keratinocytes in Melanoma Invasion/Progression

Several early studies demonstrated that the interaction between melanoma cells and surrounding keratinocytes plays an important role in melanoma invasion and relies both on cell–cell interactions and soluble factors. By using a three-dimensional model of reconstructed human skin based on an acellular de-epidermized dermis, Eves et al. [163,164] observed that skin cells (fibroblasts and keratinocytes) increased the dermal invasion of melanoma cells with no or low propensity for invasion on their own. The presence of skin cells also dramatically reduced the pigmentary behavior of cells, suggesting that skin cells can also regulate the differentiation status of melanoma cells. In a similar skin model, Van Kilsdonk et al. [165] demonstrated that melanoma cells, which were unable to penetrate the basement membrane, acquired invasive properties upon physical interaction with the stratified epidermis but not with undifferentiated keratinocytes. The invasion was associated with the production of MMP9 by keratinocytes and its activation upon the secretion of an unknown soluble factor by metastatic melanoma cells. Using a microfluidic device designed to study cell–cell cross-talk by secreted factors, Ayuso et al. showed that melanoma cells co-cultured with keratinocytes acquire a spindle-like morphology and undergo a metabolic shift, associated with changes in chemokine secretion [166].

The role of differentiated keratinocytes in melanoma progression was further reinforced by Golan et al.’s study showing that differentiated keratinocytes promote melanoma vertical invasion by activating melanoma cell Notch signaling [167].

Notch signaling is involved in skin homeostasis, melanocytic stem cells, and skin cancer. Early studies revealed that the Notch receptor expression is increased, and the Notch pathway is activated in human melanoma lesions [168,169,170,171]. By interacting with other cascades such as ERBB, Nodal, and Wnt, Notch signaling participates in the control of several aspects of melanoma pathogenesis [172], such as melanoma cell survival and proliferation [168,173,174], N-cadherin expression [173], and progression in vivo [168]. In addition, Notch signaling dysregulation contributes to modifying the tumor cytokine milieu, shaping the immunological microenvironment toward a pro-tumoral context [175].

Golan et al. [167] showed that the binding of melanoma Notch receptors with Notch ligands, Jagged1, and Delta-like-1 (DLL1), expressed by the upper epidermal layers, leads to the expression of miR-222/221 endowed with pro-invasive properties. Interestingly, in non-invasive melanoma cells, miR-222/221 transcription is repressed by MITF, which cooperates with RBP-Jκ, possibly by physical interaction [176]. Notch pathway activation and NICD translocation into the nucleus results in the removal of MITF/RBP-Jκ from the miR-222/221 promoter and in the expression of miR-222/221. MiR-222/221 directly targets growth factor receptor-bound protein 10 (GRB10) and estrogen receptor-alpha (ESR1), which participate in promoting melanoma invasion. This mechanism is supported by the analysis of clinical samples showing active Notch signaling in melanoma cells in direct interaction with DLL1-expressing keratinocytes and in the vertical growth phase. In addition, Notch-related genes were upregulated, whereas genes targeted by miR-222/221 were downregulated in invasive melanomas compared to melanoma in situ samples.

We recently unveiled how melanoma cells use sphingolipid paracrine signaling to compromise keratinocyte adhesion and invade the dermis [177].

Sphingolipids are major components of the eukaryotic membranes. Some of them, particularly the central and interconnected ceramides, sphingosine and sphingosine-1-phosphate (S1P), are also bioactive molecules [178]. Ceramides are hydrolyzed in sphingosine by acid ceramidase (AC). The phosphorylation of sphingosine by sphingosine kinase isoenzymes 1 or 2 (SK1/2) produces S1P. Mostly secreted, S1P binds to S1P receptors (S1PRs), a family of five specific G protein-coupled receptors (S1P1–S1P5) [179]. S1P can also act intracellularly by interacting with the pro-apoptotic effector BAK [180], the E3 ubiquitin ligase TRAF2 [181], the heat-shock proteins HSP90s [182], and by regulating histone acetylation [183]. Whereas ceramide mediates cell-stress responses, including cell-growth arrest, apoptosis, and senescence, S1P is usually involved in cell proliferation, cell survival, cell migration, inflammation, and angiogenesis [178]. Therefore, their dynamic interconversion acts as a major determinant in cell fate and in cancer progression [184], and it is referred to as the “sphingolipid rheostat” [185].

In the human epidermis, sphingolipid synthesis is tightly regulated and participates in several aspects of skin homeostasis [186]. Ceramides play a major role in the formation of the skin barrier and the prevention of transepidermal water [1,187]. S1P, in contrast with its effect on most cells, promotes keratinocyte growth arrest [188,189,190] and protects them from apoptosis [191,192]. It also acts as a keratinocyte chemoattractant [190,193] and enhances their differentiation [194,195,196], presumably by increasing intracellular calcium concentration [193]. S1P production by keratinocytes is increased as a response to various stresses [197,198,199]. The conversion of ceramides to S1P protects UVB-stressed keratinocytes from ceramide-induced apoptosis [200]. S1P enhances the NF-κB–C/EBP-dependent production of cathelicidin antimicrobial peptide (CAMP) upon endoplasmic reticulum (ER) stress [182,199] and induces the S1PR-dependent synthesis of pro-inflammatory cytokines upon bacterial invasion [197,198]. The accumulation of S1P associated with keratinocyte deficiency in S1P-specific phosphatase (SPP1), which dephosphorylates S1P into sphingosine, or in S1P lyase (S1PL1), which catalyzes the irreversible degradation of S1P into ethanolamine phosphate and hexadecenal, leads to altered epidermis thickness [194,201,202], enhanced keratinocyte differentiation [194,196,202], and aberrant intercellular junctions [202]. Unbalanced S1P production and dysregulation of the associated signaling pathways are also involved in inflammatory skin diseases such as psoriasis, atopic dermatitis, and itch [203].

On their side, cancers and, particularly, melanomas, display numerous dysregulations in the sphingolipid metabolism [184,204,205]. Our team and others have highlighted how such dysregulations exert protumoral effects, such as the metastatic dissemination of melanoma tumor cells via pro-invasive interactions with stromal fibroblasts [206], the transition between the proliferative and the invasive phenotype [207], immune escape [208], and the contribution to the resistance to BRAF [209] and immune checkpoint inhibitors [210].

As revealed by the expression analysis of sphingolipid metabolism enzymes in melanoma cell lines and patient sample public data, melanomas have an increased expression of the S1P-producing enzyme SK1 from primary stages [177,206,210]. In contrast, the expression of S1P-degrading enzymes is either downregulated or unchanged [177], suggesting a balance in favor of the production of S1P and supported by increased S1P levels in plasma from patients with melanomas.

Considering the overexpression of SK1 in primary melanomas and the deleterious effects of S1P accumulation on epidermis homeostasis, we investigated the role of the SK1/S1P axis in the context of early-stage cutaneous melanoma. We first observed that primary melanoma cells with high SK1 expression, associated with increased S1P secretion, have an enhanced ability for dermal invasion in human reconstructed skins and abolish the keratinocytes’ control over their motility in 2D assays. As pointed out from RNA-seq analysis of in vitro S1P-exposed keratinocytes, S1P induces a downregulation of the adhesion molecule E-cadherin, mediated by S1P2- and S1P3-receptor signaling, which increases the expression of the E-cadherin transcriptional repressors Snail and Slug. Furthermore, the effect of S1P on keratinocyte adhesion to melanoma cells and motility is positively correlated to the expression level of E-cadherin in melanoma cells. Importantly, the analysis of primary melanoma samples also showed a significantly decreased E-cadherin expression in epidermis areas neighboring tumors expressing high levels of SK1, with no change in tumor E-cadherin. We found that epidermal E-cadherin levels were inversely correlated with the Breslow thickness in early-stage tumors, indicating an association between high SK1 expression in tumors, decreased expression of proximal keratinocyte E-cadherin, and increased tumor cell invasive potential. Whereas E-cadherin loss in tumor cells is long known as a feature associated with melanoma dermal invasion [211], our study revealed an alternate mechanism promoting the invasion of E-cadherin-expressing melanoma cells via keratinocyte adhesion remodeling.

The potential of S1P to induce the downregulation of epidermal E-cadherin expression was recently confirmed in a study using human reconstructed skins formed with S1P lyase-deficient keratinocytes [202]. In contrast, S1P signaling through S1PR2 participates in the maintenance of the epidermal barrier by inducing the expression of proteins such as Zo-1, Flg2, and Cdsn [212]. These studies suggest different effects of S1P on keratinocyte adhesion, loosening cell–cell interaction in lower layers but consolidating them in upper layers. Moreover, keratinocytes, known as “cytokinocytes” [38], enhance their production of pro-inflammatory factors upon S1P exposure [198,212]. Since inflammation promotes phenotypic changes of melanoma cells [213] and modulates the immune landscape in the skin [38], how keratinocytes neighboring melanoma cells participate in early melanoma progression under S1P-subverted dialogue with tumor cells remains to be explored.

### 5.6. Role of Keratinocytes in Melanoma-Associated Inflammation

Melanoma initiation, progression, and metastasis have long been associated with chronic inflammation [214,215]. After oncogenic transformation in a permissive microenvironment, tumor cells themselves produce pro-inflammatory factors, triggering the recruitment of various stroma, inflammatory, and immune cells, which in turn fuel inflammation [216,217,218,219]. Keratinocytes also produce cytokines and chemokines, which are involved in the regulation of physiological immune responses in the skin and in the pathophysiology of skin-inflammatory diseases [38]. Nevertheless, their contribution to inflammation in the context of primary melanoma was poorly explored. A new insight into their potential involvement was recently offered by spatial approach-based studies addressing the interactions of primary melanoma within its native morphological microenvironment. 

Using spatial RNA profiling and IHC analysis of an extended cohort of human benign and tumor melanocytic lesions, including epidermal tissue, Kiuru et al. strongly identified keratinocytes in the proximity of melanoma cells as a site of enhanced expression of S100A8 and S100A9 proteins [220], confirming previous sporadic observations [221,222,223].

The S100 family Ca^2+^-binding proteins S100A8 and S100A9 form a heterodimer complex (S100A8/A9, calprotectin), which belongs to the damage-associated molecular pattern (DAMP) family (also known as alarmins) and exerts intracellular and paracrine effects by interacting with different sensors, including RAGE, TLR4, EMMPRIN, MCAM, and ALCAM [224,225]. S100A8/A9 is not expressed in normal epidermis but is strongly upregulated and released during wound healing, skin stresses, and in inflammatory skin diseases [226,227]. In keratinocytes, S100A8/A9 stimulates the production of inflammatory cytokines, including CXCL8, CXCL1, and TNFα, which in turn enhance the secretion of S100A8 and S100A9 in a positive feedback loop associated with growth stimulation [228] and induces MMPs [222]. In melanoma cells, S100A8/A9 also induces pro-inflammatory factors and MMPs [221,222]. Interestingly, in primary melanoma, S100A9 and its receptor EMPRINN co-localize at the tumor-invasive edge and in the adjacent epidermis. This pattern is associated with a loss of basement membrane, suggesting an S100A9/EMMPRIN role in melanoma dissemination through the basement membrane and extracellular matrix degradation [222]. In addition, S100A8/A9 promotes melanoma growth and cell motility, and it functions as a strong soil signal in pre-metastatic organs by attracting melanoma cells [229] through their S100A8/A9 sensors [221,222,229,230,231].

Kiuru et al. showed that S100A8/A9 expression in keratinocytes is associated with the expression of KRT17 and KRT6, two keratins upregulated in wounded skin, supporting the notion that the growth of melanoma in situ within the epidermis elicits an injury response in adjacent keratinocytes. The increased expression of S100A8/A9 in primary melanoma has previously been attributed to melanoma-infiltrating immune cells [232,233] whose abundance predicts shorter survival [233]. In contrast, the spatial analysis of in situ melanoma detected S100A8 expression only in scattered immune cells within the dermis [220], suggesting a dominant role for keratinocyte S100A8 in the very early steps of melanomagenesis. Therefore, by producing S100A8/A9 in an injury-like response triggered by melanoma cells, keratinocytes might contribute to initiating the inflammation context, leading to the recruitment of inflammatory cells, including S100A8/A9-producing cells. Nevertheless, the mechanisms remain to be elucidated. Interestingly, melanoma-secreted exosomes were shown to induce a strong expression of S100A8 and S100A9 in human primary keratinocytes, but the exosomal components triggering this effect were not identified [221].

Importantly, the increased expression of S100A8 and S100A9 was not detected in melanocytes and nevi. In melanoma, S100A8/A9 expression in adjacent keratinocyte, which is significantly associated with an invasive melanoma tumor type [220], was proposed as a valuable marker complementary to melanoma-specific antigens for the differential diagnosis of early melanoma and nevi [234].

Nearly concomitantly to Kiuru et al.’s study, Yao et al. [235] demonstrated the pro-tumoral behavior of keratinocytes on melanoma growth and metastasis via the secretion of thymic stromal lymphopoietin (TSLP). TSLP is a pro-TH2 cytokine predominantly expressed by epithelial cells and keratinocytes and participates in maintaining TH2-type homeostasis at barrier surfaces [236]. Released in response to mechanical injury, infection, inflammatory cytokines, and proteases such as trypsin and papain, TSLP acts as an “alarmin” and can condition dendritic cells to initiate Type 2 responses. The dysregulated expression of and/or signaling by TSLP has been seen in a variety of inflammatory diseases, including atopic dermatitis (AD), food-hypersensitivity reactions, asthma, and cancer [237]. The tumor-promoting function, presumably associated with the induction of a TH2-type tumor microenvironment blunting anti-tumor immunity, was reported in various cancers [237,238]. In contrast, several studies indicated an anti-tumor activity of TSLP, in particular, in skin squamous-cell carcinoma [239,240].

Using mouse-inducible melanoma models in which the expression of BRAF^V600E^ was induced and Pten inactivated selectively in melanocytes by tamoxifen administration (BRAF/PTEN mice), Yao et al. [235] observed that TSLP expression increases in the epidermis overlying melanomas concomitantly with tumorigenesis. The genetic ablation of TSLP in this background delays melanoma growth and metastasis. In contrast, the pharmacological induction of TSLP in epidermal keratinocytes promotes the formation and growth of BRAF melanoma, which normally develops slowly and accelerates the progression and metastasis of BRAF/PTEN melanoma. Moreover, the pharmacological induction of TSLP promotes B16F10 melanoma cell growth in syngenic wild-type mice, but not in TSLP^−/−^ mice, and it has no effect on melanoma growth in immunodeficient mice, demonstrating the requirement of an intact immune system for TSLP to exert its pro-tumoral effect.

The authors demonstrated that TSLP signals through TSLPR are expressed by dendritic cells and promote GATA3+ Th2 cells and GATA3+ Tregs with enhanced immunosuppressive functions on CD8+ T cells in tumor-draining lymph nodes and tumor sites. Importantly, the examination of human cutaneous primary melanoma biopsies by IHC showed TSLP expression in epidermal suprabasal layers overlying invasive melanomas without a clear association between TSLP signals and Breslow depth. TSLP was detected neither in melanoma cells nor in the dermis. Moreover, GATA3+ Tregs were found enriched in invasive primary melanoma, suggesting similar TSLP-driven immunosuppressive mechanisms in human melanomas. Therefore, this study identified TSLP as a keratinocyte-derived factor promoting melanoma progression through the remodeling of its ecosystem toward an immunosuppressive environment.

The potential interest of TSLP in melanoma care, as a prognostic factor or a pharmacological target, for instance, with anti-TSLP antibodies approved for asthma treatment, will require further evaluation.

## 6. Conclusions

The emergence of an oncogenic event, originating either from keratinocytes or melanocytes, induces important modifications in the epidermal landscape. Whether such changes are associated with the initiation of keratinocyte oncogenic transformation or the diversion of healthy keratinocyte functions toward protumoral behavior remains to be fully explored in non-melanoma skin cancers. In melanoma, tumor-adjacent keratinocytes produce injury-related and pro-inflammatory factors acting either on tumor cells themselves or their microenvironment. These phenotypic changes contribute to melanoma growth, migration, invasion, and metastasis. Knowing the role of inflammation in the phenotypic switch associated with aggressiveness, one might speculate that keratinocytes also participate in the triggering of other important events in melanoma progression, which remains to be investigated. Understanding the very early mechanisms promoting skin cancer aggressiveness in its microenvironment will offer new avenues for marker and target discovery.
